# Interfacial Modification of Macro Fiber Composites for Active Low-Frequency Vibration Suppression

**DOI:** 10.3390/ma19173693

**Published:** 2026-08-30

**Authors:** Jingjing Zhou, Zhiwei Li, Jing Zhou, Renwu Song, Shan Wu, Yong Zhu, Juan He

**Affiliations:** 1 Key Laboratory of Functional Materials and Devices for Informatics of Anhui Educational Institutions, Fuyang Normal University, Fuyang 236037, China; 202401001@fynu.edu.cn (J.Z.); 2025221217@stu.fynu.edu.cn (Z.L.); swu@fynu.edu.cn (S.W.); zyshy@fynu.edu.cn (Y.Z.); juanhe78@163.com (J.H.); 2 State Key Laboratory of Silicate Materials for Architectures, School of Materials Science and Engineering, Wuhan University of Technology, Wuhan 430070, China; 3 Sanya Science and Education Innovation Park, Wuhan University of Technology, Sanya 572024, China; 4 Anhui Engineering Research Center for Agricultural Product Quality Safety Digital Intelligence, Fuyang Normal University, Fuyang 236037, China; 202403006@fynu.edu.cn

**Keywords:** macro fiber composite, interface modification, active vibration suppression, low-frequency vibration

## Abstract

Low-frequency vibration of thin-walled materials, which are widely used in aircraft, can lead to fatal damage of aircraft components and even serious accidents. Although active vibration suppression using macro fiber composite (MFC) holds promise, the weak internal interfaces of MFC—specifically, those among the piezoelectric ceramics, the polymer matrix, and the interdigitated electrodes—seriously restrict the actuation strain and effective suppression bandwidth, limiting its engineering application under broadband aerodynamic excitation. In this work, an MFC-based self-feedback device integrating sensor and actuator is proposed. To address this, the performance bottleneck of the MFC actuator is overcome through a combined interface modification strategy combining plasma etching and dopamine-inspired modification. The interfacial modification elevates the maximum actuation strain of the MFC from 915 με to 1105 με (an increase of 20.8%). When applied to an aluminum cantilever beam, a vibration suppression ratio of 98.11% is achieved at the resonant frequency (75 Hz), and the effective suppression bandwidth (suppression ratio > 50%) reaches 77 Hz. Notably, this strategy is effective on aluminum, stainless steel, and carbon fiber substrates, as equally efficient broadband suppression is realized on all three materials. A powerful pathway is thus provided to unlock the full potential of MFC for low-frequency, broadband active vibration control in aerospace applications.

## 1. Introduction

Thin-walled structures in aircraft, such as wing skins and fuselage panels, are subjected to intense broadband aerodynamic noise and vibrational loads during flight. Among these, low-frequency components below 200 Hz are particularly critical, as they overlap with the low-order structural modes and readily induce resonant fatigue, posing serious threats to flight safety and structural integrity [1,2,3]. In modern helicopters and rotorcraft, such low-frequency vibrations are even more severe due to rotating aerodynamic excitation. To address this challenge, four main categories of methods/techniques have been developed: passive isolation/damping approaches, such as dynamic vibration absorbers and viscoelastic damping layers, which are reliable and effective at high frequencies but constrained at low frequencies by bulky added masses; semi-active control approaches [4], including magnetorheological dampers and variable stiffness/damping systems, which consume low power but suffer from insufficient robustness and typically achieve suppression ratios below 50% [5]; active control approaches, such as higher harmonic control (HHC) and individual blade control (IBC), which offer strong adaptability and high low-frequency efficiency but involve complex hardware and high costs; and piezoelectric smart structure active control, which generates counteracting forces via integrated piezoelectric actuators, offering fast response, high integration, and excellent low-frequency suppression efficiency [6,7]. However, the effectiveness of piezoelectric actuators is constrained by their limited actuation strain output, which restricts the effective suppression bandwidth. Rather than optimizing material composition or control algorithms, this work addresses this bottleneck from the perspective of interface mechanics, aiming to enhance actuation linearity and broadband suppression capability through interfacial modification.

Significant progress has been made in active vibration control using piezoelectric materials in recent years [8,9]. Among these, macro fiber composite (MFC) has demonstrated unique advantages for vibration suppression in thin-walled structures due to its combination of the high actuation capability of piezoelectric ceramics and the flexibility of polymers [10]. MFC consists of rectangular piezoelectric ceramic fibers, an epoxy resin matrix, and interdigitated electrodes [11], and operates in the d_33_ mode that exploits the piezoelectric effect along the fiber length to generate substantial actuation strains. Extensive research has been conducted on MFC-based active control applications. Ma et al. [12] employed MFC for vibration control of axially moving cantilever beams, achieving suppression ratios above 85% using Proportional-Derivative (PD) combined with fuzzy control. Gawryluk et al. [13] applied Proportional (P), Derivative (D), and Proportional-Derivative (PD) control to suppress vibrations in laminated thin-walled beams under shaker excitation, demonstrating the necessity of incorporating a first-order inertial element in finite element models. Rimašauskienė et al. [14] experimentally verified that MFC actuators exhibit significantly higher suppression efficiency at resonant frequencies than at off-resonant frequencies. Zhang et al. [15] established an active control model for geometrically nonlinear vibrations of composite laminated plates using MFC, revealing the influence of control gains and MFC placement on nonlinear jump phenomena. Zhang et al. [9] further proposed an integrated composite laminate structure with embedded MFC, achieving approximately 44.4% vibration attenuation through PID control. In terms of control algorithms, Peng et al. [16] systematically compared the performance of positive position feedback (PPF), positive velocity feedback (PVF), and positive acceleration feedback (PAF) controllers in MFC-based elastic beam suppression, demonstrating the superior overall performance of PPF controllers. Regarding sensing and other applications, Zhou et al. [17] applied MFC for energy harvesting from low-speed underwater flow, validating its low-frequency response characteristics. Kim et al. [18] systematically investigated the effects of curvature on MFC displacement and natural frequencies, proposing a rapid measurement method combining swept-frequency excitation and Fast Fourier Transform (FFT) analysis. Zhu et al. [19] fabricated high-performance MFCs based on potassium sodium niobate (KNN) lead-free piezoelectric ceramics for human motion monitoring. In previous work by this group [20], a self-feedback active vibration suppression system based on MFC was designed, in which MFC-1 served as the sensor and MFC-2 as the actuator integrated on a thin-walled composite beam, achieving a suppression ratio of 82.09% at the resonant frequency of 67 Hz and an effective suppression bandwidth (suppression ratio >50%) of 52 Hz. However, a common limitation persists across these studies: the effective suppression bandwidth remains narrow. Most studies focus primarily on achieving high suppression ratios at resonant frequencies (up to ~90%) while overlooking the effective bandwidth—a critical metric for assessing real-world service performance [7,21]—which severely restricts their engineering applicability under broadband noise environments.

The fundamental origin of this bottleneck lies in the fact that MFC is a typical multi-interface composite material whose macroscopic actuation strain depends not only on the intrinsic polarization response of the piezoelectric ceramic but also, and more critically, on the interfacial load transfer efficiency [22]—that is, whether the intrinsic strain generated by the piezoelectric fibers can be efficiently and with low loss transferred through the fiber/polymer interface to the macroscopic MFC surface. Under ideal conditions, the epoxy resin matrix serves as the intermediate medium for stress transfer, synergistically outputting the fiber strain. However, once interfacial defects such as debonding or microvoids exist, the actuation stress cannot be effectively transmitted, potentially leading to complete actuator failure [23,24,25]. Various interfacial modification strategies have been explored: Lin et al. [26] systematically investigated the effects of electrode spacing, fiber thickness, interface layer thickness, and PZT volume fraction on the actuation performance of piezoelectric fiber composites, revealing that the strain decreased from 1900 με to 700 με (a reduction of 63%) as the interface layer thickness increased from 0 to 4 μm, demonstrating an exponential attenuation effect of the interface layer on effective voltage. Pandey and Arockiarajan [27] established a finite element model of MFC under thermo-electromechanical coupling based on Kirchhoff and Mindlin plate theories, finding that the nonlinear temperature distribution through the thickness significantly influences actuation performance. Zhou et al. [28] introduced a porosity correction factor to improve the electric fatigue model of MFC, demonstrating that fatigue life can exceed 10^6^ cycles when porosity is below 0.7%. Jin et al. [29] achieved self-biased magnetoelectric coupling by incorporating magnetic Terfenol-D particles into the epoxy adhesive layer of PZT MFC/Metglas magnetoelectric composites, constructing a magnetic gradient interface. Zheng et al. [30] established an improved shear-lag model incorporating radial roughness, revealing the opposite effects of roughness on stress transfer at different interface stiffness levels. Nevertheless, most of these studies focus on the characterization and passive protection of interfacial defects. Systematic studies that start from the mechanical essence of interfacial stress transfer and actively engineer interfaces to enhance MFC actuation linearity and broadband suppression performance remain scarce.

This study departs from the conventional mindset of “material composition optimization” and instead addresses the interfacial stress transfer mechanism, proposing a combined interface modification strategy combining plasma etching and dopamine-inspired modification to enhance the integrated vibration suppression performance of MFC. On one hand, theoretical analysis and experimental investigation are conducted to improve the interfacial bonding of MFC, thereby enhancing its ferroelectric and actuation properties. On the other hand, an integrated self-feedback suppression device that combines sensing and actuation is designed to achieve effective broadband vibration suppression of thin-walled structures under low-frequency vibration environments. The results not only enhance the actuation performance of MFC through improved stress transfer efficiency but also demonstrate good applicability on three typical aerospace thin-walled materials (aluminum, stainless steel, and carbon fiber), achieving broadband effective suppression across these substrates. This work provides new insights and methodologies for vibration suppression in thin-walled structures for aerospace applications.

## 2. Materials and Methods

### 2.1. Active Vibration Suppression Structure Design

To achieve superior vibration suppression performance over a broadband low-frequency range, this study builds upon previous findings [20] and employs a multifunctional macro fiber composite (MFC) self-developed in our laboratory that integrates sensing and actuation capabilities for vibration suppression in aircraft thin-walled structures. As a composite sheet material combining piezoelectric ceramic fibers and a polymer matrix, MFC offers both the high actuation capability of ceramics and the flexibility of polymers. Owing to the piezoelectric properties of the ceramic phase, MFC can not only convert mechanical vibration signals into electrical signals for substrate vibration detection but also transform electrical signals into mechanical stress to generate counteracting forces at the same frequency for canceling noise in the target structure.

**Figure 1:**
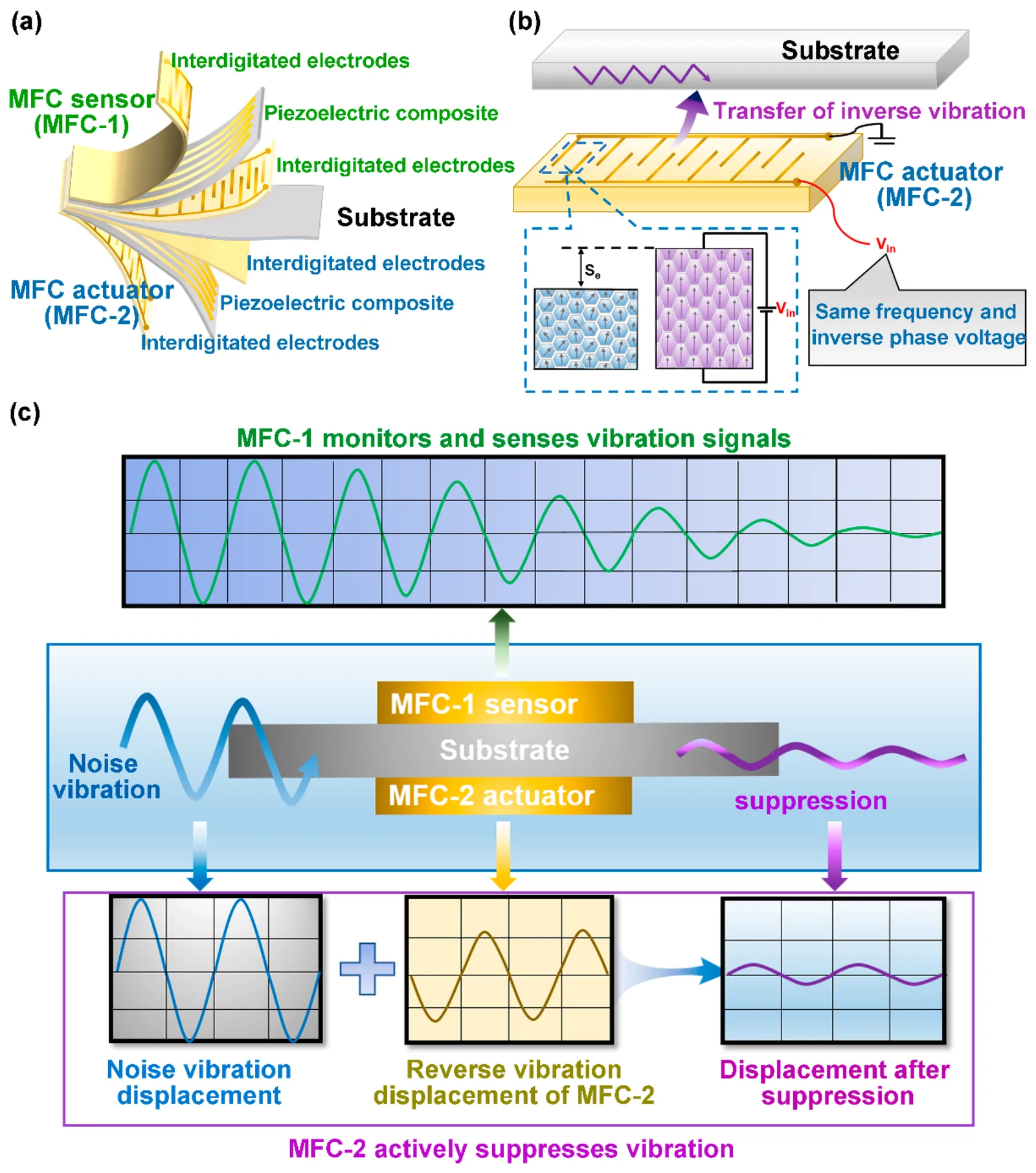
Integrated active vibration suppression system based on macro fiber composite (MFC): (**a**) MFC structure; (**b**) active vibration suppression mechanism; (**c**) design of the integrated active vibration suppression system.

As shown in Figure 1a, the functional composite layer of MFC has overall dimensions of 40 mm (length) × 6 mm (width) × 0.3 mm (thickness). The internal piezoelectric fiber composite layer is composed of PZT-5H piezoelectric ceramic strips alternately arranged with epoxy resin filler strips of equal width. The PZT-5H fibers exhibit a piezoelectric coefficient d_33_ = 750 pC/N, relative dielectric constant εᵣ = 3200, density ρ = 7500 kg/m^3^, and electromechanical coupling coefficient Kp = 0.68 (detailed properties are summarized in Table S1 of the Supplementary Information). The epoxy resin, DP460 (3M Company, St. Paul, MN, USA), is a thermosetting bisphenol A epoxy mixed at a resin-to-curing-agent ratio of 2:1 by weight, with an elastic modulus E_m_ = 3 GPa. Each individual fiber measures 40 mm × 0.3 mm × 0.3 mm, and the MFC has a PZT fiber volume fraction of approximately 50%. The MFC multifunctional sheet is sandwiched between two gold interdigitated electrode layers (thickness = 0.5 μm, electrode spacing = 0.3 mm), forming a complete MFC device. The substrate, made of aluminum alloy 6061-T6 with dimensions of 80 mm × 15 mm × 0.5 mm, serves as the thin-walled structural analog and is attached with MFC sheets on both its upper and lower surfaces. The upper MFC sheet functions as the sensor (MFC-1), while the lower sheet serves as the actuator (MFC-2).

When vibrational noise is transmitted to the substrate, MFC-1 detects the vibration signal and converts it into an electrical signal, which is processed by the active control algorithm and then sent to MFC-2 as a counter-phase driving signal. Upon receiving this signal, MFC-2 generates an alternating electric field between its interdigitated electrodes, causing domain switching and inducing macroscopic expansion and contraction of the piezoelectric fibers. This electromechanical response produces a mechanical stress with the same frequency and opposite phase to cancel the vibrational noise on the substrate, as illustrated in Figure 1c.

### 2.2. Interfacial Stress Transfer Mechanism

Although the above-designed structure can achieve effective vibration suppression of thin-walled materials, experiments revealed that the consistency of MFC materials is poor and the piezoelectric functionality remains far from fully realized. This is primarily because MFC is a material with multiple interfaces and complex architecture; the presence of interfacial debonding or other anomalies can prevent effective transmission of the actuation stress and may even lead to complete actuator failure. The interface issue thus constitutes a major factor restricting the development of MFC.

**Figure 2:**
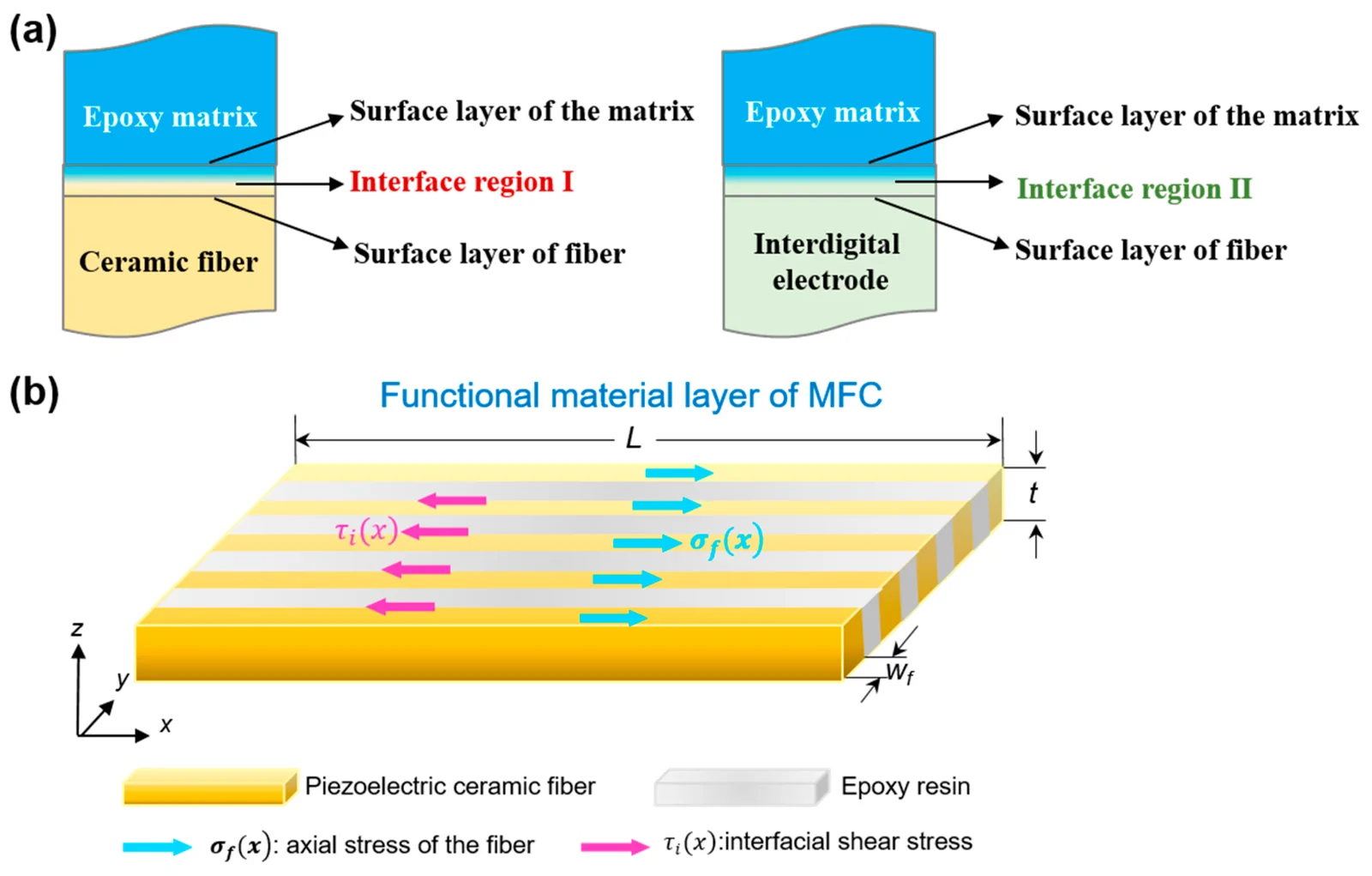
(**a**) Interface structure of MFC; (**b**) schematic illustration of the functional composite layer of MFC.

The interfaces in MFC mainly include the interface between piezoelectric ceramic fibers and the polymer matrix, as well as the interface between the interdigitated electrodes and the polymer matrix, as shown in Figure 2. Among these, the interface between the piezoelectric ceramic and epoxy resin has the most significant influence on the actuation performance. As a flexible polymer, epoxy resin enhances the material’s flexibility, enabling the MFC to be applied to curved surfaces and other complex operating conditions. When MFC functions as an actuator, the epoxy resin is “clamped” by the piezoelectric fiber strips on both sides, allowing it to participate with the fibers in the actuation process, making the epoxy resin the primary medium for stress transfer. Additionally, epoxy resin is a strong insulating material that imposes a substantial voltage division on the applied voltage. For the interface between the piezoelectric composite layer and the interdigitated electrodes, under ideal conditions, they are intimately bonded; however, in practice, an adhesive is required to bond the two layers during fabrication. To avoid introducing a new interface, the same polymer material as used in the piezoelectric composite layer (epoxy resin) is typically employed as the adhesive. In this configuration, the interface issue between the piezoelectric composite layer and the interdigitated electrodes transforms into the interface problem among the piezoelectric composite layer, the polymer layer, and the interdigitated electrode layer.

#### 2.2.1. Interfacial Shear Stress Transfer Model

The cross-section of MFC exhibits a periodic laminated structure (Figure 2), with piezoelectric fiber strips and epoxy resin strips alternately arranged with equal widths along the width direction. Taking a differential element of a single rectangular fiber of thickness t and width $w_{f}$ (Figure 2b), and based on one-dimensional stress equilibrium and shear-lag theory [22,31,32], the variation in the axial stress of the fiber, $\sigma_{f}(x)$, equals the resultant force of the interfacial shear stress $\tau_{i}(x)$ over the perimeter in magnitude, with the opposite direction

(1) $$\frac{d\sigma_{f}(x)}{dx}=-\frac{2\left(w_{f}+t\right)}{w_{f}t}\tau_{i}(x)$$

where the negative sign indicates that the drag force of the interfacial shear stress on the fiber acts opposite to the direction of fiber elongation. With the fiber dimensions $h_{f}$ = $w_{f}$ = 0.3 mm, this simplifies to

(2) $$\frac{d\sigma_{f}(x)}{dx}=-\frac{4}{h}\tau_{i}(x).$$

Assuming linear elastic behavior of the interface layer with thickness δ (much smaller than the fiber thickness t), the interfacial shear stress is proportional to the axial relative displacement between the fiber and the matrix

(3) $$\tau_{i}(x)=\frac{G_{i}}{\delta }\left[u_{f}(x)-u_{m}(x)\right]$$

where $G_{i}$ is the equivalent shear modulus of the interface layer, and $u_{f}(x)$ and $u_{m}(x)$ are the axial displacements of the piezoelectric fiber and the adjacent epoxy resin strip, respectively.

Introducing the elastic constitutive equation for the piezoelectric fiber

(4) $$\sigma_{f}(x)=E_{f}\varepsilon_{el}(x)=E_{f}(\varepsilon_{f}(x)-\varepsilon_{p})$$

where $E_{f}$ is the elastic modulus of the piezoelectric ceramic. In this equation, the total strain $\varepsilon_{f}(x)$ consists of the elastic strain $\varepsilon_{el}(x)$ and the piezoelectric eigenstrain $\varepsilon_{p}$ (i.e., the electric-field-induced free strain). The elastic strain is the portion that contributes to the stress, while the eigenstrain represents the actuation strain generated by the electric field. Thus, the stress is governed by the elastic strain component rather than the total strain.

Combining Equations (2) and (4), and defining the relative displacement $s(x)=u_{f}(x)-u_{e}(x)$, with the matrix strain $\varepsilon_{m}=u_{e}^{\prime }(x)$ considered constant along the fiber length, a second-order governing equation for s(x) is obtained

(5) $$\frac{d^{2}s}{dx^{2}}-\beta^{2}s=0$$

where the characteristic parameter β is defined as

(6) $$\beta^{2}=\frac{4G_{i}}{E_{f}\cdot w_{f}\cdot \delta }$$

which has the dimension of (length)^−2^, so β has the dimension of (length)^−1^ (1/m), ensuring that the stress transfer characteristic length $L_{c}=1/\beta$ is properly defined $L_{c}$, the reciprocal of β, has the dimension of length and characterizes the characteristic decay distance of interfacial shear stress from the fiber end toward the interior (the stress transfer characteristic length $L_{c}$).

For a piezoelectric fiber with free ends, the boundary conditions are $\sigma_{f}(-L/2)=\sigma_{f}(L/2)=0$. Substituting the general solution of Equation (5) and combining with Equation (4), the distribution of axial stress along the fiber length is obtained

(7) $$\sigma_{f}(x)=E_{f}\varepsilon_{p}\left[1-\frac{\cosh(\beta x)}{\cosh(\beta L/2)}\right].$$

The macroscopic output strain $\varepsilon_{eff}$ is defined as the average of the fiber elastic strain $\varepsilon_{el}(x)=\sigma_{f}(x)/E_{f}$ along the fiber length from *x* = −*L*/2 to x = *L*/2

(8) $$\varepsilon_{eff}=\frac{1}{L}\int_{-L/2}^{L/2}\varepsilon_{el}(x)dx.$$

Substituting Equation (7) into Equation (8) and integrating yields

(9) $$\varepsilon_{eff}=\varepsilon_{p}\left[1-\frac{\tanh(\beta L/2)}{\beta L/2}\right].$$

The stress transfer efficiency η defined as the ratio of the macroscopic output strain $\varepsilon_{eff}$ to the intrinsic strain $\varepsilon_{p}$, is then

(10) $$\eta =\frac{\varepsilon_{eff}}{\varepsilon_{p}}=1-\frac{\tanh(\beta L/2)}{\beta L/2}.$$

From Equation (10), it can be seen that when βL≫1 (the fiber length is much greater than the stress transfer characteristic length), η→1, and the efficiency coefficient η approaches its maximum value of 1 (100%). Consequently, η is already close to its theoretical maximum. This conclusion indicates that interfacial modification does not increase strain by substantially increasing the absolute value of η, but rather by significantly suppressing microscopic interfacial slip and nonlinear hysteresis, thereby enabling the polarization-enhanced intrinsic strain to be transferred to the macroscopic surface of MFC in a highly linear manner—this is the underlying physical mechanism for the experimentally observed increase in actuation strain and the significant improvement in S-E curve linearity. The significant stiffness mismatch between the PZT fibers ($E_{p}\approx 60\;\mathrm{GPa}$) and the epoxy matrix ($E_{m}\approx 3\;\mathrm{GPa}$) plays a critical role in force and kinetic energy transfer within the MFC. The stiffness ratio of approximately 20:1 means the high-stiffness fibers carry the overwhelming majority of the axial load, while the low-stiffness matrix primarily serves for load transfer and stress redistribution. This mismatch amplifies the importance of interfacial quality: adequate bonding enables efficient strain energy transfer, while insufficient bonding exacerbates stress concentration and promotes debonding.

It should be noted that the above shear-lag model is based on linear elasticity, which applies to intact interfaces. For interfacial defects such as microcracks and debonding, a fracture mechanics criterion is introduced in Section 2.2.2 to characterize the resulting nonlinear degradation. Beyond the strain transfer described by the shear-lag model, the overall vibration suppression process involves a multi-stage energy transfer chain: the electromechanical strain generated by the PZT fibers is first transferred to the epoxy matrix through the fiber/matrix interface; the matrix then distributes the strain to the overall MFC laminate, producing an equivalent actuation force and bending moment; the MFC subsequently transfers the actuation force to the host structure through the adhesive bond layer; and the host structure responds dynamically based on its modal characteristics and boundary conditions. This comprehensive framework bridges the microscopic actuation mechanism with the macroscopic vibration suppression performance. Although the epoxy adhesive layer between the piezoelectric functional layer and the interdigitated electrodes does not directly participate in in-plane stress transfer, its presence produces a dual effect. On one hand, upon curing, it imposes a normal compressive stress on the vertical sidewalls of the PZT fibers (i.e., along the width direction of the fibers, as illustrated in Figure 2b), increasing the frictional resistance at the vertical sidewall interfaces, which is equivalent to introducing an additional stiffness term into the shear constitutive Equation (3), thereby enhancing the effective interfacial shear modulus

(11) $$G_{eff}=G_{i}+\mu \frac{\sigma_{n}}{\gamma }$$

where $G_{eff}$ is the effective interfacial shear modulus, representing the actual interfacial shear stiffness after modification, which combines the intrinsic shear modulus of the interface layer and the additional stiffness contribution from enhanced friction; $G_{i}$ is the intrinsic shear modulus of the interface layer, reflecting the inherent shear deformation resistance of the interface material itself; μ is the friction coefficient; $\sigma_{n}$ is the normal compressive stress; and γ is the shear strain. Considering the curing shrinkage of the epoxy resin (~3–5%) and its elastic modulus (~3 GPa), together with stress relaxation during curing and the geometric constraint of the periodic laminated structure, the residual normal compressive stress $\sigma_{N}$ on the fiber sidewalls is estimated to be on the order of 10–15 MPa. This value is significantly lower than the theoretical full-field shrinkage stress (~90–150 MPa), which is a reasonable consequence of the localized nature of the sidewall constraint and the stress relaxation within the compliant matrix. On the other hand, the epoxy adhesive layer uniformly transfers the electric field from the interdigitated electrodes to the fibers, preventing non-uniform strain induced by local electric field distortion.

It should be noted that Equation (11) is an engineering approximation used to illustrate the contribution of frictional resistance ($\mu \frac{\sigma_{n}}{\gamma }$) to the effective interfacial shear stiffness, rather than a rigorous constitutive relation. Furthermore, the individual parameters in Equation (11), including $G_{i}$ and $G_{eff}$, have not been directly measured in this study; therefore, the model is presented as a mechanistic framework to support interpretation of the experimental trends rather than as quantitative experimental proof of the proposed interface-strengthening mechanism.

#### 2.2.2. Mechanical Criterion for Interface Debonding

When microcracks or voids exist at the vertical sidewall interfaces, the stress concentration at the fiber ends is significantly amplified. For the dominant mode II (sliding-mode) interfacial debonding in MFC, the stress intensity factor $K_{II}$ at the crack tip is related to the average interfacial shear stress τ and the crack length a by

(12) $$K_{II}=Y\tau \sqrt{\pi a}$$

where Y is a dimensionless geometric factor (for rectangular fibers, Y≈1.12 [33]).

When $K_{\mathrm{II}}\geq K_{\mathrm{IIC}}$ (where $K_{\mathrm{IIC}}$ is the interfacial fracture toughness), the crack undergoes unstable propagation, leading to complete interfacial debonding. Following debonding, $G_{i}$ in Equation (3) drops sharply to the residual friction level. Although η in Equation (10) does not change substantially owing to the long fiber length, the interfacial slip introduces nonlinear hysteresis that significantly degrades the linearity and response speed of strain output.

### 2.3. Interfacial Modification and Fabrication

Based on the analysis of the above physical model, this study adopts a combined interfacial modification strategy combining “plasma treatment and dopamine bio-inspired modification”. Plasma treatment is performed using a plasma cleaner to process the material surface. The treatment parameters are as follows: O_2_ atmosphere, pressure of 50 Pa, RF power of 100 W, treatment distance of 5 cm, and treatment duration of 10 min. The dopamine solution has a concentration of 2 mg/mL with a pH of 8.5 adjusted by Tris buffer, and the samples are immersed at room temperature for 24 h, followed by rinsing with deionized water three times. On one hand, it cleans the surface by removing surface contaminants. On the other hand, it impels ionized gases (such as O_2_) onto the material surface, forming reactive functional groups. This treatment has minimal impact on the material surface while improving the interfacial bond strength. In this study, plasma treatment removes weak boundary layers and introduces oxygen-containing reactive functional groups, increasing the effective interfacial contact area, which is equivalent to reducing the effective interface thickness δ in Equation (3) and thus enhancing the interfacial bonding strength and effective interfacial stiffness.

Additionally, the application of modifiers significantly influences interfacial bonding. In 2007, Lee et al. [34] reported in Science that dopamine can self-polymerize under weakly alkaline conditions to form polydopamine (PDA), which is rich in catechol groups capable of forming strong coordination bonds on inorganic material surfaces. The PDA layer formed by dopamine self-polymerization establishes covalent bridges between the inorganic piezoelectric ceramic fibers and the organic epoxy resin, simultaneously enhancing $G_{i}$ in Equation (3) and $K_{\mathrm{IIC}}$ in Equation (12). After modification, the intrinsic strain $\varepsilon_{p}$ increases due to enhanced polarization, while the linearity of interfacial transfer is also improved; these two effects collectively contribute to the significant increase in macroscopic actuation strain. The modified MFCs are assembled as the sensor (MFC-1) and actuator (MFC-2), respectively, to comprehensively evaluate the effect of interface optimization on electromechanical coupling performance.

**Figure 3:**
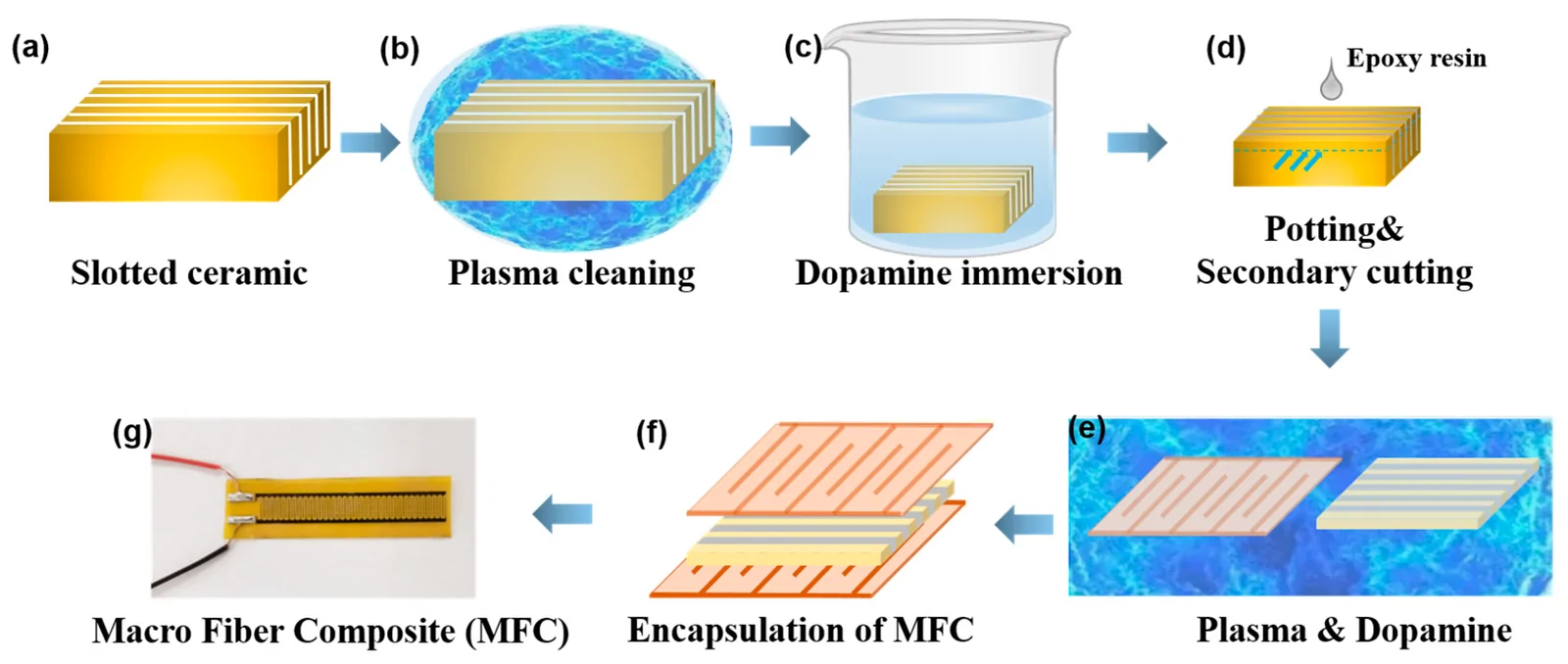
Preparation of modified interface-based MFC: (**a**) cutting and slotting of piezoelectric ceramic; (**b**) plasma cleaning of piezoelectric ceramic; (**c**) dopamine immersion of piezoelectric ceramic; (**d**) pouring epoxy resin and secondary cutting; (**e**) re-surface treatment of the interdigital electrode and the piezoelectric composite sheet; (**f**) encapsulation; (**g**) Macro Fiber Composite (MFC).

MFCs are fabricated using a two-step cutting method. The fabrication process with interface modification is shown in Figure 3. First, the PZT-5H piezoelectric ceramic is grooved, and the grooved ceramic skeleton is cleaned with a plasma cleaner under vacuum conditions for 10 min. The sample is then immersed in a dopamine solution for 24 h, removed, and dried. Epoxy resin DP460 is poured into the grooved skeleton and cured at room temperature for 24 h. Secondary cutting yields composite sheets composed of piezoelectric ceramic and epoxy resin. After the composite sheet and interdigitated electrodes are subjected to the combined Plasma and Dopamine interface treatment, gold interdigitated electrodes are applied. Subsequently, the MFCs are poled under an electric field of 2 kV/mm at 120 °C for 30 min. Then, the entire assembly is encapsulated with polyimide (PI) to obtain the interface-optimized MFC. The fabricated MFCs are equipped as MFC-1 sensors and MFC-2 actuators on the thin-walled structure (substrate), completing the fabrication of the self-feedback integrated active vibration suppression device.

**Figure 4:**
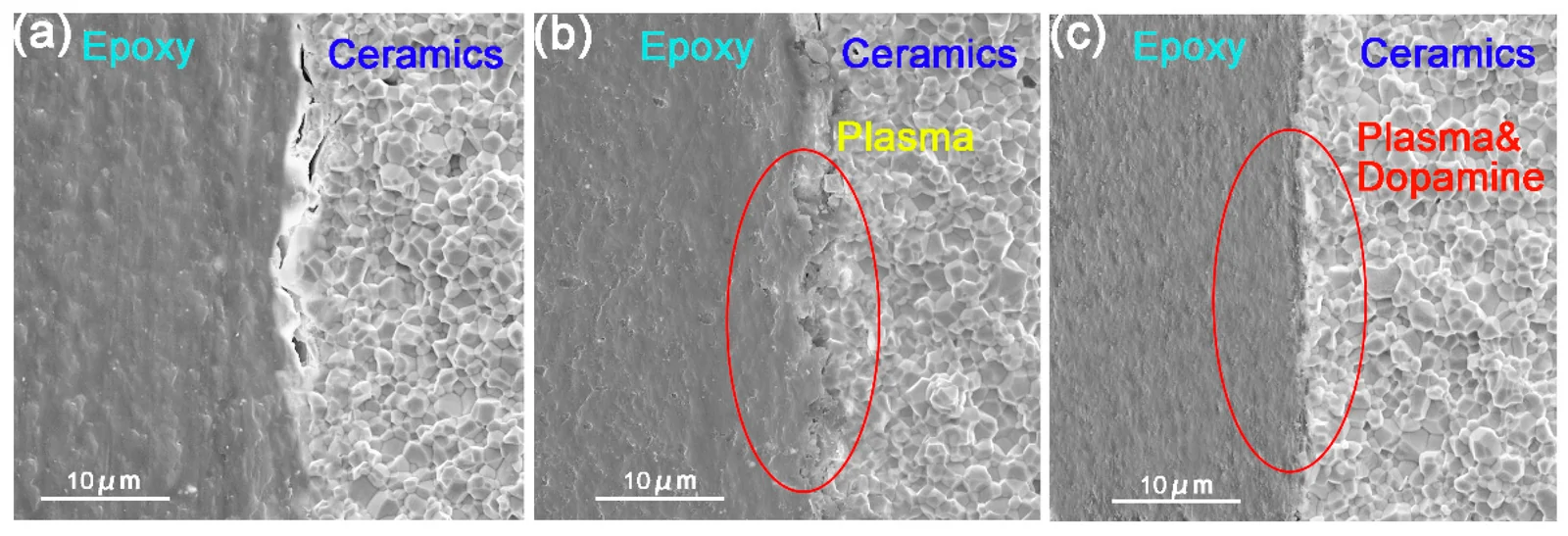
Cross-sectional SEM images of the MFC interface under different treatment conditions: (**a**) untreated; (**b**) plasma-treated; (**c**) combined plasma and dopamine-treated.

To directly visualize the effect of the combined interface modification on interfacial morphology, cross-sectional SEM images of the MFC interfaces were obtained (Figure 4). As shown in Figure 4a, the untreated sample exhibits distinct voids and debonding at the ceramic/epoxy interface, indicating poor interfacial adhesion. After plasma treatment alone, as shown in Figure 4b, the interfacial gap is reduced but some micro-cracks remain, suggesting that plasma treatment provides only partial improvement. After the combined plasma and dopamine treatment (Figure 4c), the interface becomes well-bonded with no visible gaps. Notably, a thin dark layer is observed at the interface, which is attributed to the polydopamine (PDA) coating formed by dopamine self-polymerization. The PDA layer is deposited at the nanoscale (thickness < 20 nm) and does not significantly affect the interface layer thickness. The enhanced adhesion is proposed to be associated with the formation of coordination bonds and hydrogen bonds between the catechol groups of PDA and the inorganic PZT surface, as well as covalent bridges with the epoxy resin, as previously reported in the literature [34]. However, direct chemical evidence for such bonding is not provided in the present study.

## 3. Experiments

The signal acquisition and processing control circuit hardware consists of two main components: a signal conditioning and acquisition system and a digital signal processing system. The former handles signal conditioning, acquisition, and output, including input and output modules. The latter performs A/D and D/A conversion control, data transmission, control algorithm execution, and Ethernet data exchange, with a core processing chip (OMAP-L137, Texas Instruments Inc., Dallas, TX, USA) and total system power consumption below 30 W. A shaker (SCU-200, Suzhou Sushi Testing Instrument Co., Ltd., Suzhou, China) is used to simulate vibrational noise environments. When the substrate mounted on the shaker vibrates, MFC-1 attached to the upper surface of the substrate senses the vibration signal through the piezoelectric effect, converting the driving force signal into a voltage signal of the same frequency and transmitting it to the controller. After processing by a PID algorithm (with controller parameters P = 2.5, I = 0.8, D = 0.3), the controller outputs a driving voltage with the same frequency but a 180° phase shift relative to the vibration signal. This signal is amplified to 1000 V sinusoidal waves using a high-voltage amplifier (YE5873A, Trek, Inc., Lockport, NY, USA) and applied to MFC-2 on the lower surface of the substrate. Upon receiving the electrical signal, MFC-2 generates a driving force with the same frequency and opposite phase as the vibrational noise based on the inverse piezoelectric effect, thereby achieving active vibration suppression. The displacement measurement accuracy is ±2%, and the voltage measurement accuracy is ±1%, with a frequency resolution of 1 Hz.

During testing, metal foil strain gauges (BE120-3A, AVIC Electrical Measurement Instrument (Xi’an) Co., Ltd., Xi’an, China) are attached along the axial and transverse directions of the MFC in a bridge configuration to measure strain responses. A laser sensor (MTS-025, MTI Instruments, Inc., Albany, NY, USA) is used to monitor the vibration displacement amplitude at the free end of the substrate.

### 3.1. Effect of Interface Optimization on MFC Performance

**Figure 5:**
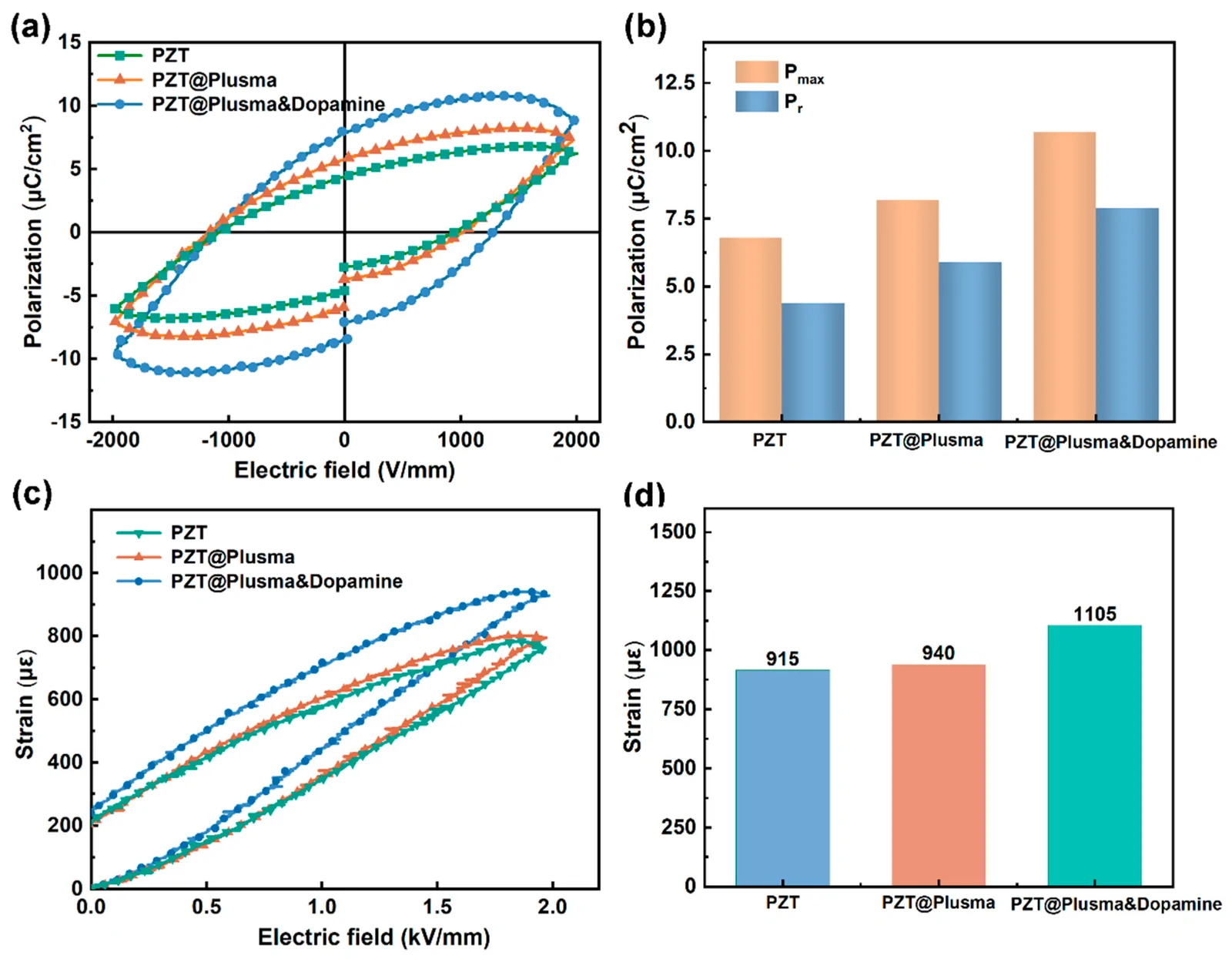
Comparison of ferroelectric and strain properties before and after MFC interface treatment: (**a**) P-E curve; (**b**) polarization intensity change; (**c**) S-E curve; (**d**) strain change. Data are presented as mean ± SD (*n* ≥ 5). Statistical significance was determined by one-way ANOVA followed by Tukey’s HSD post hoc test.

After interfacial modification, the interfacial bond strength of the MFC is significantly enhanced, with both ferroelectric and actuation properties effectively improved. All quantitative data in this section are reported as mean ± standard deviation (SD), where SD represents the standard deviation of individual measurements around their mean. Each reported value is based on at least 5 independently fabricated specimens (*n* ≥ 5). For comparisons among the three treatment groups (untreated, plasma-treated, and combined plasma–dopamine treated), one-way analysis of variance (ANOVA) was performed, followed by Tukey’s HSD (Honestly Significant Difference) post hoc test for pairwise comparisons. Statistical significance was set at *p* < 0.05. Figure 5a,b show the ferroelectric hysteresis loops (P-E curves) and polarization intensity changes in MFC under different interface treatment conditions. It can be seen that plasma treatment alone slightly improves the ferroelectric properties, while the combined Plasma and Dopamine treatment yields a more pronounced enhancement.

Specifically, after plasma treatment alone, the remanent polarization $P_{r}$ of MFC increases from 6.9 ± 0.3 to 7.8 ± 0.4 μC/cm^2^ (*n* = 5, *p* < 0.05), and the maximum polarization $P_{\max}$ increases from 8.9 ± 0.4 to 10.4 ± 0.5 μC/cm^2^ (*n* = 5, *p* < 0.05). The underlying physical mechanism is that plasma treatment first introduces oxygen-containing reactive functional groups and is expected to enhance surface reactivity, which is consistent with the observed improvement in interfacial bonding. Subsequently, the polydopamine (PDA) layer formed by dopamine self-polymerization establishes strong chemical bonding between the inorganic PZT-5H and organic epoxy resin via catechol groups. This combined effect significantly enhances the interfacial bond strength, effectively inhibiting the initiation of microvoids and other defects at the interface, thereby enhancing the charge output capability and ferroelectric response. The enhanced interfacial bonding suppresses microvoid initiation and local electric field distortion during poling, thereby allowing a more uniform and complete polarization process, thus the observed increase in remanent polarization is reasonably attributed to more complete domain switching enabled by improved interfacial bonding, rather than changes in intrinsic ferroelectric properties. Additional characterization such as dielectric spectroscopy or leakage current measurements would further strengthen this interpretation, and we have identified this as a direction for future work in Section 4.

Furthermore, the effect of interface modification on MFC actuation strain is evaluated through unipolar strain-electric field (S-E) curve measurements, with results shown in Figure 5c,d. The unmodified PZT-based MFC exhibits a maximum output strain of 915 ± 8 με (*n* = 5, *p* < 0.05). After plasma treatment alone, the maximum strain increases to 940 ± 7 με (*n* = 5, *p* < 0.05). Following combined Plasma and Dopamine modification, the maximum strain further increases significantly to 1105 ± 10 με (*n* = 5, *p* < 0.01). The S-E curves in Figure 5c reveal that the untreated sample exhibits a pronounced “pinching” phenomenon in the low-field region (0–1 kV/mm), characterized by sluggish strain response and inward concavity—a typical signature of nonlinear hysteresis. After the combined plasma and dopamine treatment, this pinching phenomenon becomes noticeably less pronounced, and the S-E curve becomes highly linear (R^2^ > 0.99), indicating effective suppression of nonlinear hysteresis. The hysteresis-loop areas (the area between the loading and unloading branches of the unipolar S-E curves) are approximately 384.2 με·kV/mm for the untreated sample, 409.2 με·kV/mm for the plasma-treated sample, and 433.6 με·kV/mm for the combined plasma–dopamine treated sample. The larger absolute hysteresis area of the combined treatment reflects the significantly increased actuation strain range (from 746 με to 931 με) rather than increased nonlinear hysteresis. This indicates that interface modulation not only enhances the ferroelectric response but also substantially improves the actuation output capability.

Notably, the increase in actuation strain (~20.8%) significantly exceeds the increase in remanent polarization (~13.0%), suggesting that the strain improvement does not originate solely from polarization enhancement. According to the shear-lag model established in Section 2.2 (Equation (10)), for the long fibers with L = 40 mm used in this study, βL≫1, and the stress transfer efficiency η already approaches its theoretical upper limit. Therefore, the room for interface modification to improve the absolute value of η is extremely limited. Consequently, the physical origin of the strain enhancement lies in the fact that the combined Plasma and PDA modification significantly suppresses microscopic interfacial slip and nonlinear hysteresis, enabling the polarization-enhanced intrinsic strain $\varepsilon_{p}$ to be transferred to the macroscopic MFC surface in a highly linear manner with negligible loss. This inference is highly consistent with the significant improvement in the linearity of the S-E curve in Figure 5c, where the modified curve is smooth and the “pinching” phenomenon in the low-field region (i.e., the nonlinear behavior characterized by sluggish strain response and inward concavity at low electric fields) becomes much less pronounced. The improved linearity of actuation strain ensures that the actuator can still precisely output the corresponding counter-phase signal when deviating from the resonant frequency or when the required driving amplitude changes, thereby laying the foundation for the subsequent enhancement of suppression capability and bandwidth.

### 3.2. Performance of Interface-Optimized MFC

To achieve low-frequency active vibration suppression based on MFC, MFC-1 must possess excellent sensing sensitivity to precisely detect weak vibration signals, while MFC-2 must exhibit highly linear actuation response to output control forces strictly in phase opposition to the vibration signals. In this section, the sensing performance of MFC-1 and the actuation performance of MFC-2 are systematically characterized.

**Figure 6:**
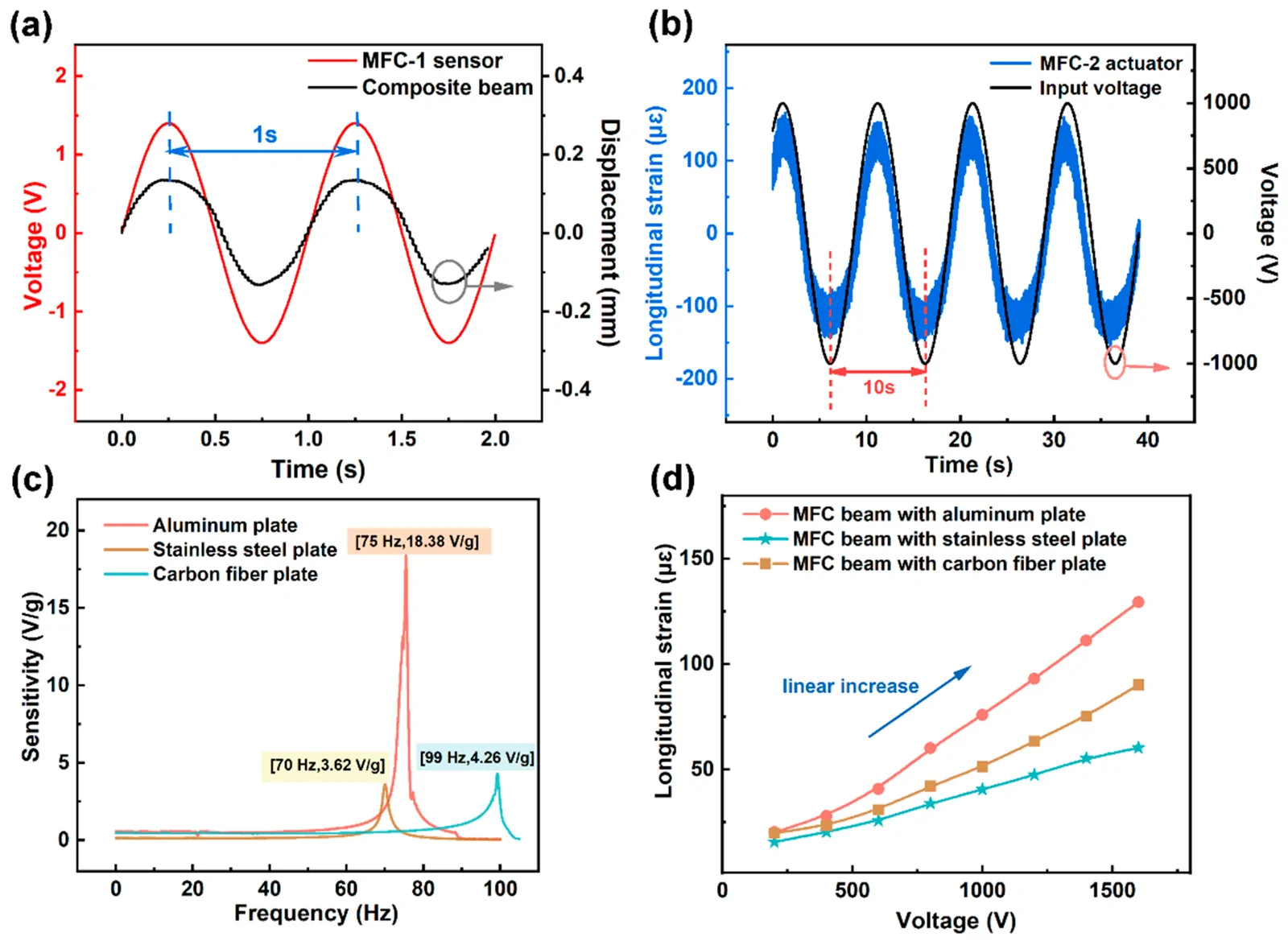
Characterization of the integrated active vibration suppression device: (**a**) sensing voltage of MFC-1 versus drive displacement of the composite beam at 1 Hz excitation; (**b**) drive deformation of MFC-2 in response to the input control voltage; (**c**) sensing sensitivity of MFC-1 with different materials; (**d**) linear relationship between the axial strain of MFC-2 and the applied driving voltage.

First, the vibration sensing capability of MFC-1 is evaluated. A vibrational noise signal at a frequency of 1 Hz is applied to the composite beam, and the sensing output voltage of MFC-1 and the beam driving displacement are recorded. The results are shown in Figure 6a. The sensing voltage signal (red curve) and the displacement response signal (black curve) exhibit identical peak/valley positions and frequencies, indicating that MFC-1 can accurately detect the specific frequency vibration signals in the substrate.

Regarding actuation performance, the vibration signal sensed by MFC-1 is processed by the PID controller and converted into a voltage signal with the same frequency and opposite phase, which is then input to MFC-2, generating the corresponding inverse piezoelectric driving deformation. Figure 6b shows the temporal comparison between the input voltage (black curve) and the MFC-2 driving deformation (blue curve), demonstrating excellent frequency and phase synchronization, satisfying the actuation requirements for active suppression.

To verify the adaptability of MFC-1 to different substrate materials, MFC-1 is attached to aluminum, stainless steel, and carbon fiber plates—three typical thin-walled structural materials used in aerospace applications—and the sensing sensitivity at their respective resonant frequencies is measured. The results are shown in Figure 6c. At the resonant frequency of 75 Hz for the aluminum substrate, the sensing sensitivity reaches 18.38 V/g (=1.87 V/(m/s^2^)). The sensitivities for the stainless steel substrate (resonant frequency 70 Hz) and carbon fiber substrate (resonant frequency 99 Hz) are 3.62 V/g and 4.26 V/g, respectively. These results indicate that the MFC-1 sensor achieves effective vibration detection on all tested substrates, with the highest sensitivity observed on aluminum, which is attributed to better mechanical impedance matching with the piezoelectric ceramic.

Figure 6d further presents the length-direction strain response of MFC-2 under different driving voltages. As the driving voltage increases linearly from 0 to 1000 V, the output strain exhibits approximately linear growth with a linearity fit R^2^ > 0.99, confirming excellent voltage-strain linear response characteristics. This linearity ensures that MFC-2 can precisely output counteracting forces corresponding to target vibrations under various amplitude conditions, thereby establishing the actuation foundation for broadband vibration suppression.

### 3.3. Vibration Suppression Performance After Interface Optimization

**Figure 7:**
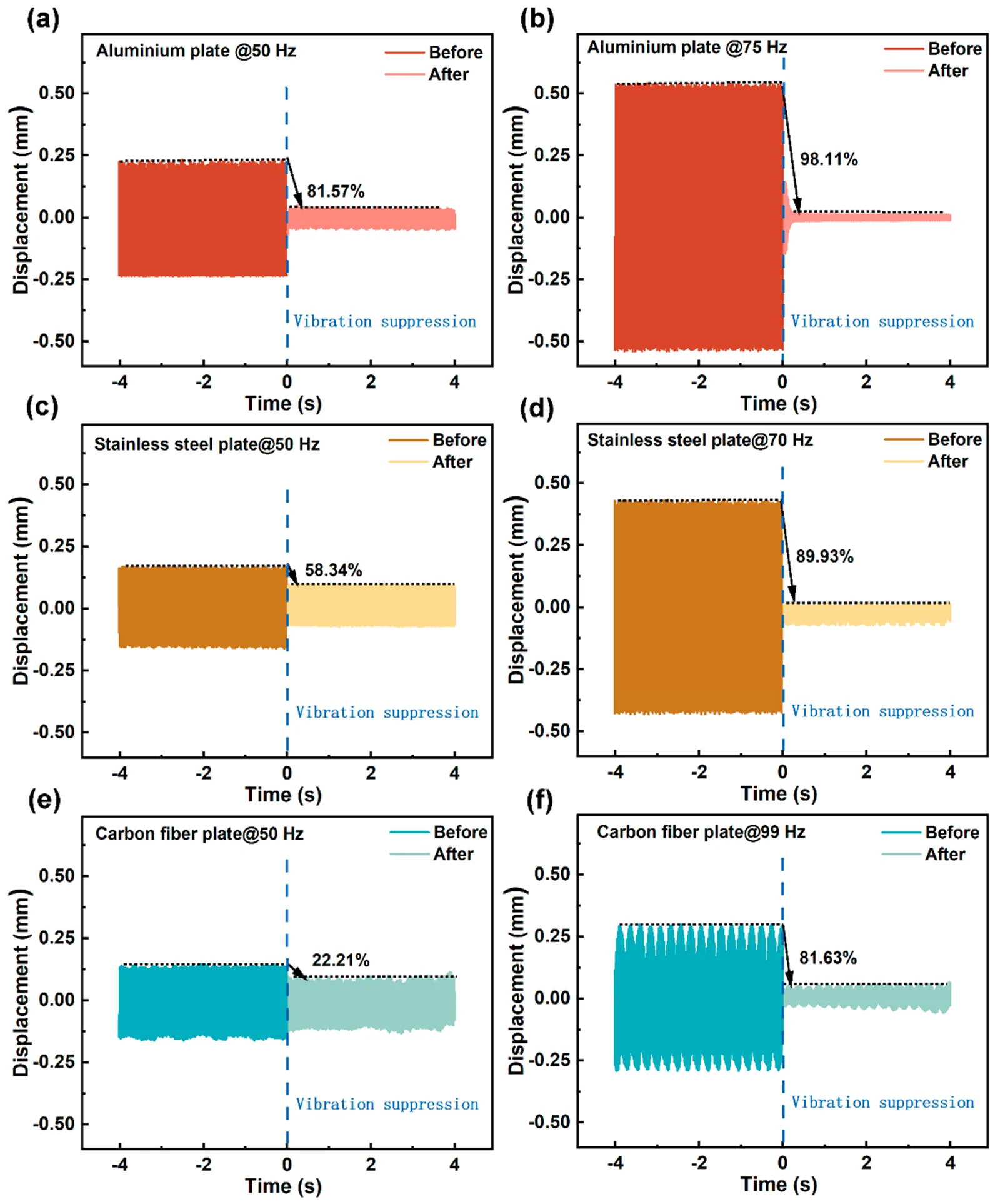
Vibration suppression performance of the MFC active control system on different substrate materials: (**a**) aluminum plate achieves 81.57% suppression at 50 Hz (non-resonant); (**b**) aluminum plate achieves 98.11% suppression at 75 Hz (resonant); (**c**) stainless steel plate achieves 58.34% suppression at 50 Hz (non-resonant); (**d**) stainless steel plate achieves 89.93% suppression at 70 Hz (resonant); (**e**) carbon fiber plate achieves 22.21% suppression at 50 Hz (non-resonant); (**f**) carbon fiber plate achieves 81.63% suppression at 99 Hz (resonant).

The vibration suppression effectiveness is closely related to the resonant characteristics of the composite beam, while the improvement in actuation strain linearity resulting from interface modification provides the foundation for suppression performance when deviating from resonance. In this study, the suppression performance of MFC-2 under both resonant and off-resonant conditions is systematically evaluated. Aluminum, stainless steel, and carbon fiber plates are used as substrates under a vibrational noise excitation of 5 m/s^2^. The suppression ratio is calculated by comparing the substrate displacement amplitude before and after activating MFC-2, with results shown in Figure 7.

The suppression ratio was calculated using the following equation:

(13) $$\eta =\frac{A_{uncontrolled}-A_{controlled}}{A_{uncontrolled}}\times 100\%$$

where $A_{uncontrolled}$ and $A_{controlled}$ are the displacement amplitudes at the free end of the beam before and after activating the MFC actuator, respectively.

The highest suppression efficiency for all three substrates occurs at their respective resonant frequencies—75 Hz for aluminum, 70 Hz for stainless steel, and 99 Hz for carbon fiber—with suppression ratios reaching 98.11%, 89.93%, and 81.63%, respectively. Away from the resonant frequencies, the suppression ratio decreases, though the rate of decay varies with substrate material. Notably, under identical off-resonant excitation (50 Hz), the suppression ratios differ substantially among the three substrates: aluminum maintains 81.57%, stainless steel achieves 58.34%, while carbon fiber reaches only 22.21%.

This discrepancy can be explained from multiple perspectives. First, mechanical impedance matching is one important factor: as a force source, the efficiency of drive energy injection from the MFC actuator into the substrate depends on the degree of mechanical impedance matching between the two. Aluminum (elastic modulus E = 70 GPa, density ρ = 2.7 g/cm^3^) has acoustic impedance relatively close to that of PZT-5H piezoelectric ceramic (E = 60 GPa, ρ = 7.5 g/cm^3^), enabling efficient transfer of actuation strain to the substrate structure. In contrast, the carbon fiber substrate (E = 130 GPa, ρ = 1.6 g/cm^3^) exhibits significant impedance mismatch, causing partial reflection of drive energy at the interface and consequently reducing suppression efficiency. However, this impedance mismatch alone does not fully explain the relatively low suppression ratio of the carbon fiber substrate at 50 Hz, where additional factors such as higher modal stiffness, lower intrinsic damping, and weaker modal coupling between the MFC actuator and the carbon fiber’s structural modes may also contribute. Beyond impedance matching, several additional factors also contribute to the observed differences in suppression performance across substrates. Second, the modal coupling between the MFC actuator and the substrate plays a critical role—when the MFC’s dominant actuation mode is well matched with the substrate’s low-order bending modes, the energy injection efficiency is substantially enhanced. Third, the placement of the MFC actuator on the substrate significantly affects the control authority; in this study, all MFC actuators were bonded near the fixed end of the cantilever beam, where the bending strain is maximized, thereby ensuring optimal actuation efficiency. Fourth, the properties of the epoxy adhesive layer—including its shear modulus, bonding strength, and thickness—directly influence the transfer efficiency of the actuation strain from the MFC to the substrate; any defects such as voids or partial debonding within the adhesive layer would introduce additional energy dissipation and reduce the effective driving force delivered to the substrate. Fifth, the intrinsic damping characteristics of the substrate materials themselves also affect the overall vibration suppression performance, as higher internal damping leads to faster dissipation of vibrational energy, thereby influencing the measured suppression ratios. This result also suggests that optimizing MFC dimensions and layup design for different substrate materials is an important direction for further enhancing universality.

Furthermore, the aluminum substrate maintains a high suppression ratio of 81.57% even at off-resonant frequencies, which is directly related to the improvement in actuation strain linearity resulting from interface modification (Section 3.1). According to the shear-lag model analysis in Section 2.2, the combined Plasma and Dopamine modification effectively suppresses the nonlinear hysteresis in the S-E curve of MFC-2 (Figure 5c), enabling the actuator to maintain accurate voltage-strain linear response when deviating from resonance and when the required driving voltage amplitude varies with excitation conditions. This is further verified in Figure 6d, where MFC-2 strain increases linearly with driving voltage (R^2^ > 0.99). This high linearity is considered to be a key factor that allows MFC-2 to output precisely matched counter-phase driving forces under off-resonant conditions, thereby broadening the effective suppression frequency range.

In summary, the improvement in actuation linearity from interface modification and impedance matching optimization together provide a reasonable explanation for the broadband suppression capability of this system—the former ensures that MFC-2 can maintain a linear output of counter-phase forces when frequency detuning occurs, while the latter determines the baseline efficiency of drive energy injection into different substrates. It should be noted that these interpretations are derived from the modified shear-lag model and indirect experimental observations (such as S-E loop linearity improvement and reduced hysteresis area); direct experimental verification of interfacial shear modulus enhancement and complete elimination of microscopic slip would require further interfacial characterization and mechanical testing. The superposition of these two mechanisms enables the active suppression system to exhibit excellent broadband suppression performance across the three typical aerospace thin-walled materials, laying a solid foundation for its engineering applications.

## 4. Results and Discussion

**Figure 8:**
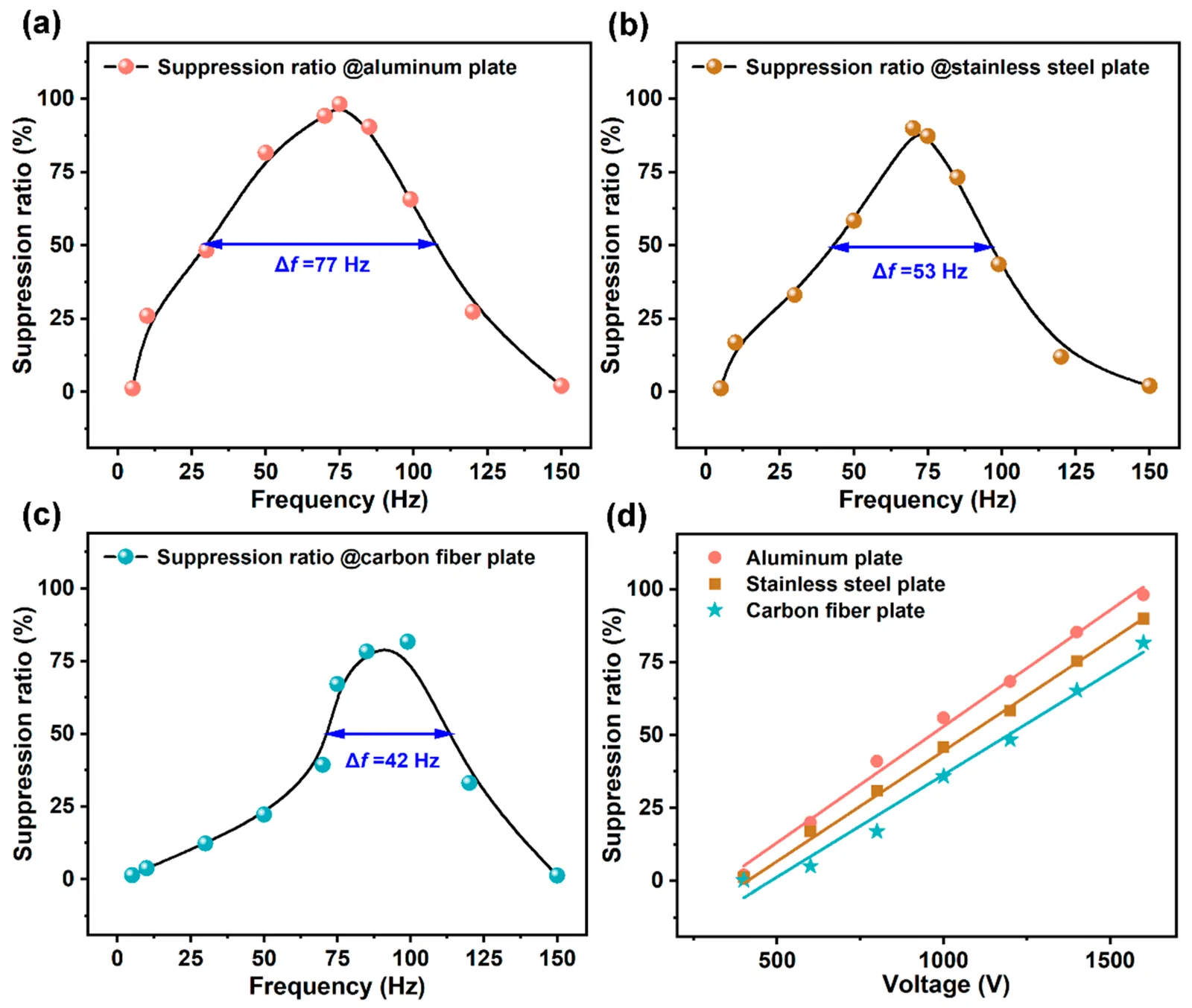
MFC-based active vibration suppression system realizes low-frequency broadband vibration suppression (**a**) Δf = 77 Hz for aluminum plate, (**b**) Δf = 53 Hz for stainless steel plate, (**c**) Δf = 42 Hz for carbon fiber plate, (**d**) the vibration suppression ratio increases linearly with the increase in driving voltage (red line for aluminum plate, brown line for stainless steel plate, and green line for carbon fiber plate).

The vibrational disturbances encountered during aircraft fuselage service are not single-frequency but distributed over a broad frequency range (typically 1–200 Hz). Therefore, focusing solely on the suppression performance at the resonant frequency is insufficient to assess the actual suppression capability of the system; the effective suppression bandwidth is the key metric for evaluating broadband suppression performance. In this study, the effective bandwidth is defined as the frequency range over which the suppression ratio exceeds 50%. Based on the data shown in Figure 7, further measurements yield the suppression ratio–frequency curves for the three substrates, with results presented in Figure 8. The frequency-response curves were obtained by measuring the suppression ratio at discrete frequency points across the range of 30–120 Hz. At each frequency point, the measurement was repeated more than five times to ensure reliability, and the suppression ratio was determined from the converged values. The curves in Figure 8 were generated by connecting these discrete data points, which provides a reliable characterization of the broadband suppression performance. The effective bandwidth boundaries were determined directly from the measured frequency points at which the suppression ratio first exceeded 50% and last remained above 50%; no interpolation was applied between adjacent measurement points. The frequency resolution of the measurements is 1 Hz, so the reported boundaries are accurate to within ±1 Hz.

The effective bandwidths for the aluminum, stainless steel, and carbon fiber substrates are 77 Hz, 53 Hz, and 42 Hz, respectively. For the aluminum substrate, the peak suppression ratio at the resonant frequency (75 Hz) reaches 98.11%, with the effective bandwidth covering a broad range from approximately 35 Hz to 112 Hz. Compared with previous work by this group (aluminum substrate suppression ratio of 82.09% with a bandwidth of 52 Hz), the interface modification employed in this work improves the effective bandwidth by an absolute value of 25 Hz (relative increase of 48.1%), while the peak suppression ratio increases by 16 percentage points (from 82.09% to 98.11%). Notably, apart from the interface modification, all other experimental variables—including MFC geometry, substrate dimensions, actuator placement, adhesive conditions, controller parameters, excitation amplitude, and measurement configuration—were kept consistent with our previous work [20].

This significant improvement can be interpreted as combined effects at two levels: first, the combined Plasma and Dopamine modification enhances the interfacial shear modulus and fracture toughness, significantly suppressing microscopic interfacial slip and nonlinear hysteresis, thereby maintaining a highly linear voltage-strain response of MFC-2 across the entire operating voltage range (Figure 6d). Second, the high linearity actuation ensures that when deviating from the resonant frequency and when the required driving amplitude varies with the excitation frequency, the actuator can still precisely output the corresponding counteractive force, thus substantially broadening the effective suppression range from near resonance to a much wider frequency band. This interpretation is consistent with the trend shown in Figure 8d, where the suppression ratios for all three substrates increase approximately linearly with driving voltage (fit goodness R^2^ > 0.99)—a strong indication of the suppression of nonlinear hysteresis through interface modification.

Notably, the differences in bandwidth among the three substrates (aluminum 77 Hz > stainless steel 53 Hz > carbon fiber 42 Hz) are consistent with the off-resonant suppression ratio differences (Figure 7), and this result correlates with the core factor of substrate mechanical impedance matching. With acoustic impedances of approximately 17 MRayl for aluminum, 46 MRayl for stainless steel, and 9 MRayl for carbon fiber (compared with ~30 MRayl for PZT-5H), the acoustic impedance of aluminum is closest to that of PZT-5H, resulting in the highest drive energy injection efficiency; the carbon fiber substrate exhibits the largest impedance mismatch, causing partial reflection of drive energy at the interface and correspondingly narrower suppression bandwidth. This result indicates that optimizing MFC geometry and layup design for different substrate materials is an effective approach to further enhancing system universality.

In summary, the interface modification-based integrated active suppression system proposed in this paper achieves low-frequency broadband and high-efficiency suppression on all three typical aerospace thin-walled materials. Compared with previously reported studies [8,11,12,13,14,15,19], this work demonstrates a significant advantage in the critical metric of effective bandwidth—not only achieving 77 Hz broadband suppression on aluminum substrates but also providing a systematic quantification of bandwidth differences across three typical substrate materials (aluminum, stainless steel, and carbon fiber), offering experimental evidence and theoretical reference for the combined design of piezoelectric composite interface engineering and active suppression systems. In the broader context of MFC interface engineering, previous studies have largely focused on optimizing structural parameters such as electrode spacing, fiber thickness, and interface layer thickness [25,26], while systematic strategies to actively engineer interfaces for enhanced actuation linearity remain scarce. Furthermore, although plasma treatment [30,31] and polydopamine coatings [32] have been individually applied to improve surface properties, their combined application for MFC interfacial modification has not been previously explored. Our work bridges these gaps by proposing a combined interface modification strategy that combines plasma etching and dopamine-inspired modification, which not only enhances the actuation strain but also significantly improves the linearity and broadband suppression capability of MFCs. It should be noted that the present experimental design compares untreated, plasma-only, and combined plasma–dopamine treated MFCs, but does not include a dopamine-only control group. Therefore, while the combined treatment shows significantly better performance than plasma-only treatment, the individual contribution of dopamine and the existence of a true synergistic effect cannot be quantitatively separated. Future work employing a complete four-group experimental design (untreated, plasma-only, dopamine-only, and combined treatment) would be valuable to further quantify the individual and synergistic contributions of each modification step.

## 5. Conclusions

This study addresses the demand for low-frequency (<200 Hz) broadband vibration suppression in aircraft thin-walled structures, focusing on the critical bottleneck of limited actuation strain output and restricted suppression bandwidth in MFC multi-interface composites caused by insufficient interfacial bonding. A combined interface modification strategy combining “plasma etching and dopamine-inspired modification” is proposed, and systematic investigations from theoretical modeling, experimental validation, to system integration are conducted. The specific conclusions are as follows:

(1) A modified shear-lag model adapted to the periodic laminated structure of rectangular fibers in MFC is established. It is revealed that when Lβ >> 1, the stress transfer efficiency η already approaches its theoretical upper limit; therefore, the core contribution of interface modification is not to increase the absolute value of η, but rather to significantly suppress microscopic slip and nonlinear hysteresis by enhancing the effective interfacial bonding (as reflected in the improved S-E loop linearity and reduced hysteresis area), thereby enabling the polarization-enhanced intrinsic strain to be transferred to the macroscopic surface with high linearity. This finding provides a clear theoretical direction for MFC interface engineering.

(2) The combined modification increases the remanent polarization from 6.9 to 7.8 μC/cm^2^ and the maximum actuation strain from 915 to 1105 με (an increase of 20.8%), while the “pinching” phenomenon in the low-field region of the S-E curve is significantly reduced. Benefiting from this linearity improvement, the integrated active suppression system achieves high-efficiency suppression on all three typical aerospace thin-walled materials: the aluminum substrate attains a peak suppression ratio of 98.11% at the resonant frequency of 75 Hz with an effective bandwidth (suppression ratio > 50%) of 77 Hz, representing an absolute improvement of 25 Hz (relative increase of 48.1%) compared with the previous group work of 52 Hz. The stainless steel and carbon fiber substrates achieve peak suppression ratios of 89.93% and 81.63%, with effective bandwidths of 53 Hz and 42 Hz, respectively. The suppression ratios for all three substrates increase linearly with driving voltage, verifying the system’s engineering tunability and its good applicability on three typical aerospace thin-walled materials, though its broader universality requires further validation.

In summary, by providing a mechanistic framework for understanding interfacial stress transfer, this work establishes a physical–chemical mechanistic framework from “interface modification” to “strain linearity enhancement” and then to “broadband suppression performance breakthrough.” The SEM characterization provided in this study has offered direct micro-morphological evidence for the improved interfacial contact. However, it should be noted that, due to current experimental limitations, the complete understanding of the interfacial strengthening mechanisms—particularly the chemical bonding effects—remains primarily based on indirect experimental evidence and theoretical analysis. Future work employing direct chemical characterization techniques such as XPS and FTIR would be valuable to further reveal the interfacial chemical bonding mechanisms and to more comprehensively validate the proposed physical–chemical framework. The developed shear-lag model and combined interface modification strategy not only provide theoretical tools and process references for piezoelectric composite interface engineering but also demonstrate a promising pathway that does not rely on the development of new materials but rather breaks through device performance bottlenecks through interfacial modulation of existing material systems. This concept can be extended to other piezoelectric stacks, flexible electronics, and structural health monitoring applications, offering a “structure–function integration” solution for intelligent vibration mitigation of lightweight thin-walled structures under complex vibrational environments.

## Data Availability

The original contributions presented in this study are included in the article/Supplementary Material. Further inquiries can be directed to the corresponding author.

## Supplementary Materials

The following supporting information can be downloaded at: https://www.mdpi.com/article/10.3390/ma19173693/s1, Figure S1: Schematic illustration of (a) construction and operation mode of MFC, (b) MFC sample; Figure S2: SEM of PZT-5H piezoelectric ceramic; Figure S3: Platform building (1–Displacement sensor; 2–Thin-walled composite beam; 3–Accelerometer; 4–Strain gauge; 5–Vibration exciter; 6–PC; 7–Control board; 8–Power amplifier; 9–Digital voltmeter); Figure S4: Principal diagram of vibration suppression logic diagram; Table S1: Technical data of PZT-5H piezoelectric ceramic.
