# Supplementary material for: Interfacial Modification of Macro Fiber Composites for Active Low-Frequency Vibration Suppression
Source: Materials (Basel). 2026 Aug 30;19(17):3693. doi: 10.3390/ma19173693 (PMC13566986; doi:10.3390/ma19173693)
Supplement: Supplementary file 1 [file materials-19-03693-s001.zip › materials-4475841-supplementary.pdf]

materials

MDPI

Supplementary Material of Article

Interfacial Modification of Macro Fiber Composites for Active Low-Frequency Vibration Suppression

Jingjing Zhou [1,†], Zhiwei Li [1,†], Jing Zhou [2,3,*], Renwu Song [4], Shan Wu [1], Yong Zhu [1] and Juan He [1]

[1] Key Laboratory of Functional Materials and Devices for Informatics of Anhui Educational Institutions, Fuyang Normal University, Fuyang 236037, China; 202401001@fynu.edu.cn (J.Z.); 2025221217@stu.fynu.edu.cn (Z.L.); swu@fynu.edu.cn (S.W.); zyshy@fynu.edu.cn (Y.Z.); juanhe78@163.com (J.H.)
[2] State Key Laboratory of Silicate Materials for Architectures, School of Materials Science and Engineering, Wuhan University of Technology, Wuhan 430070, China
[3] Sanya Science and Education Innovation Park, Wuhan University of Technology, Sanya 572024, China
[4] Anhui Engineering Research Center for Agricultural Product Quality Safety Digital Intelligence, Fuyang Normal University, Fuyang 236037, China; 202403006@fynu.edu.cn
* Correspondence: zhoujing@whut.edu.cn
† These authors contributed equally to this work.

1. Materials preparation

The MFC consists of rectangular piezo ceramic rods sandwiched between layers of adhesive, electrodes, and polyimide film, as shown in Fig. S1 (a). MFC was fabricated by the cutting-filling method. The process includes cutting, casting, secondary cutting, packaging, and polarisation. PZT-5H piezoelectric ceramic and DP460 (3M Company , St. Paul, MN, USA) were used to prepare piezoelectric fiber composite sheets. The gold interdigital electrode was etched on polyimide film (Shenzhen Hongxiang Circuit Technology Co., Ltd., Bao'an District, Shenzhen, China) as an electrode layer that transfers the applied voltage directly to the ribbon-shaped rods.

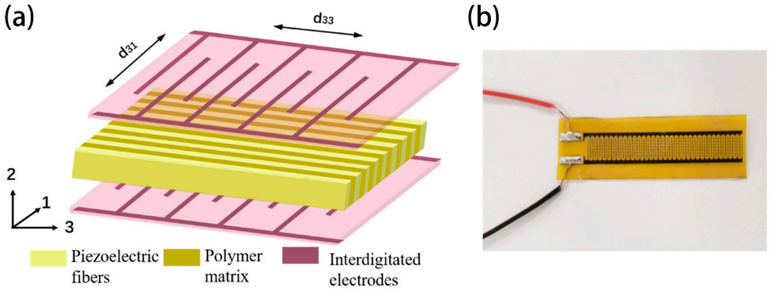

Figure S1. Schematic illustration of (a) construction and operation mode of MFC, (b) MFC sample.

Table S1. Technical data of PZT-5H piezoelectric ceramic.

Parameter	Symbol	Units	Value
Piezoelectric coefficient	d_{33}	pC/N	750
Density	ρ	Kg/m^3	7500
Coupling coefficient	K_p		0.68
Dielectric constant (1 kHz)	ε_r		3200
Dielectric loss factor (1 kHz)	tanδ		2%

The technical data of PZT-5H piezoelectric ceramic is shown in Table S1. The SEM of PZT-5H is present in Fig. S2. The data shows that PZT-5H has a high piezoelectric con-

stant, high electromechanical coupling coefficient, and low dielectric loss factor. In addition, the tensile properties of the PZT-5H fibers were measured to evaluate the structural integrity of the fibers under operating conditions. The ultimate tensile strain of PZT-5H was found to be in the range of approximately 1500 to 2000 με. Since the maximum actuation strain of the MFC in this work is 1105 με, which is well below this ultimate value, the PZT fibers operate within a safe range.

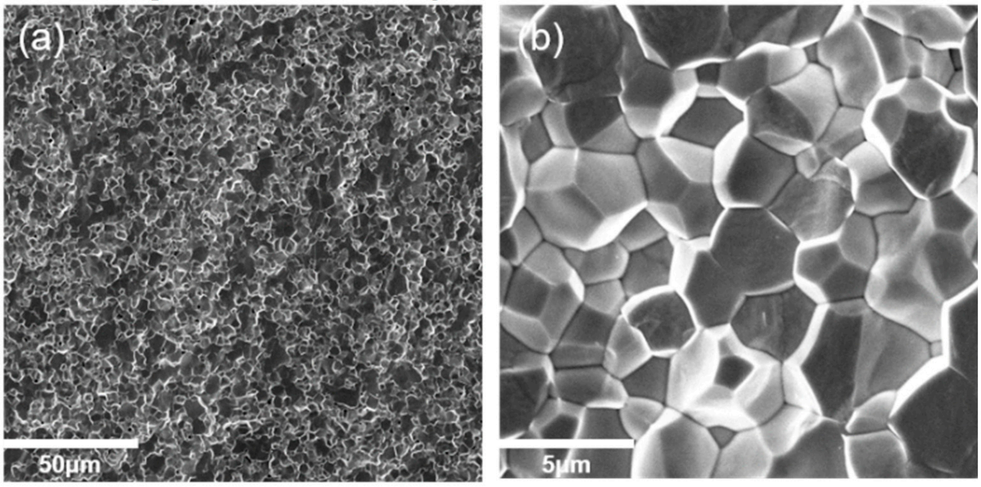

Figure S2. SEM of PZT-5H piezoelectric ceramic

2. Design of experiment station

The vibration control system (Fig. S3) developed consisted of the MFC composite beam, a vibrostand (SCU-200, Suzhou Sushi Testing Instrument Co., Ltd., Suzhou, China), a digital voltmeter (7510, Tektronix, Inc., Beaverton, OR, USA), a laser displacement sensor (MTS-025, MTI Instruments, Inc., Albany, NY, USA), a control board, and a power amplifier. In performing the experimental analysis, the tip displacement of the thin-walled composite beam was investigated and presented in the article. The noise vibrations of the composite beam were caused by the impact of a vibrostand.

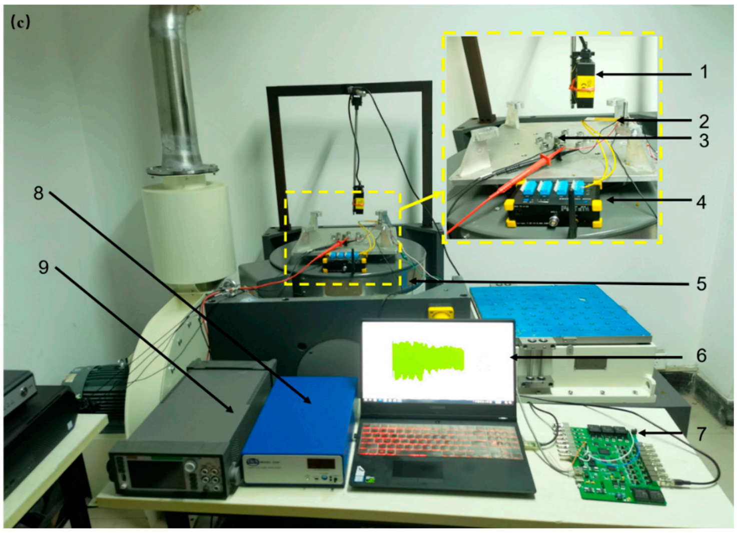

Figure S3. Platform building (1–Displacement sensor; 2–Thin-walled composite beam; 3–Accelerometer; 4–Strain gauge; 5–Vibration exciter; 6–PC; 7–Control board; 8– Power amplifier; 9–Digital voltmeter)

The driving displacement of MFC can achieve vibration suppression when the driving response of MFC is the same as the input frequency of external noise, and the phase difference is 180°. The vibration suppression algorithm is constructed, as shown in Fig.

S4, based on the abovementioned principle. When the external noise vibration signal a (t) is generated, the MFC sensor converts the driving signal into the voltage signal U (t). The acquisition and control chip is used to determine the voltage signal U (t) threshold value. If the judgment result is negative, then the chip stops signal processing. Suppose the judgment result is positive that the chip will produce the starting signal with the same frequency and phase difference of 180°; after that, the trigger signal is amplified by the power amplifier and then input into the MFC drive as the driving voltage signal V (t). If the MFC actuator converts the electrical signal into the driving signal b (t) with the same frequency and phase difference of 180°, it is negative feedback to the vibration signal a (t). Lastly, the vibration signal becomes a (t)-b (t) to realize vibration suppression. The MFC-1 sensor continuously monitors the process of vibration suppression and feedback the force and vibration suppression effect of the composite beam in the form of voltage amplitude.

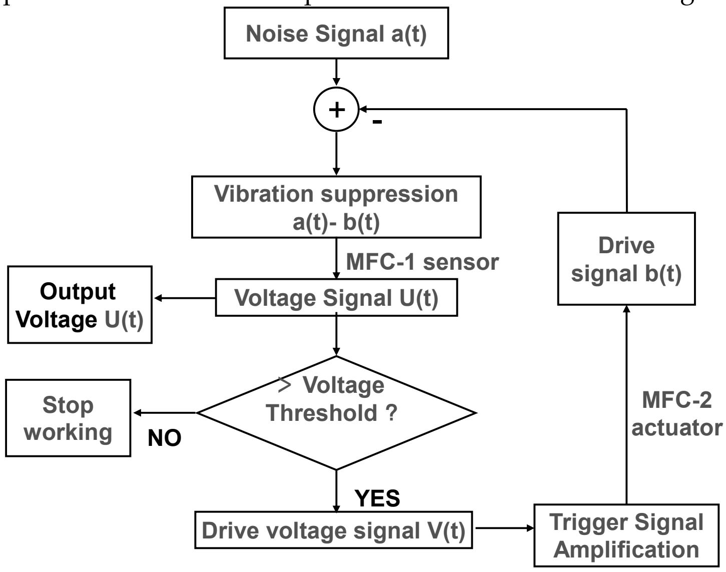

Figure S4. Principal diagram of vibration suppression logic diagram.
